# How risky is caring for emergency patients at risk of malpractice litigation: a population based epidemiological study of Taiwan's experiences

**DOI:** 10.1186/1472-6963-9-168

**Published:** 2009-09-17

**Authors:** Che-Ming Yang, Shin-Han Tsai, Wen-Ta Chiu

**Affiliations:** 1 Taipei Medical University School of Health Care Administration, Taipei, Taiwan, Republic of China; 2 Taipei Medical University Graduate Institute of Injury Prevention and Control, Taipei, Taiwan, Republic of China; 3 Taipei Medical University-Shuang Ho Hospital, Taipei, Taiwan, Republic of China

## Abstract

**Background:**

Emergency medicine has generally been considered a high risk specialty. The purpose of this study is to assess the risk of being sued in the district courts for caring emergency room (ER) patients from the perspective of epidemiology.

**Methods:**

This research was designed to be a retrospective population based cohort study. We intended to find out the incidence of litigations arising from ER patients and that of birth inpatients in Taiwan, and computed their relative risks. The inclusion criterion was set to be incidents transpired in the time period of 1998 to 2002. The study materials included the reimbursement claim dataset of the National Health Insurance from 1998 to 2002, and the district court decision database of the Judicial Yuan from 1999 to 2006.

**Results:**

The average annual incidence rate of becoming a plaintiff for ER patients is 0.86 per million, and for birth patients is 33.5 per million. There is a statistically significant difference between birth patients and ER patients. The relative risk comparing ER patients against birth inpatients is 0.03.

**Conclusion:**

The findings of this population based study indicate that the patient population emergency physicians are facing in Taiwan have relatively lower risks of developing litigation in comparison with the patients that come to give birth. Due to the large volume of ER patients, malpractice still pose a major threat in the emergency department, and misdiagnosis remains the major complaint of plaintiffs in subsequent litigations.

## Background

In a joint study conducted by the Harvard School of Public Health and Columbia Law School, the key informants identified six specialties that are at high risk of litigation [1]. Emergency medicine is one of the six, accompanied by neurosurgery, general surgery, orthopedic surgery, obstetrics/gynecology and radiology.

Emergency medicine, with its limited time for patient encounters, unpredictable flow, and lack of a continuing patient-physician relationship, is a high-risk field in terms of medical liability; in a retrospective study of all closed malpractice claims involving emergency physicians insured by the Illinois State Medical Inter-insurance Exchange, the researchers analyzed the data covering the 10-year period 1995 to 2004 and found out that emergency physicians with malpractice suits can expect resolution of the case to take over 45 months after an alleged incident, and their malpractice insurer will incur over 14,000 U.S. dollars in expenses regardless of the suit outcome [2]. In another study conducted in Massachusetts which has the fourth-highest median malpractice settlement payments for all states in the U.S. [3], 549 malpractice insurance claims filed against emergency physicians from 1975 to 1993 were reviewed [4]. The claims were grouped into two diagnostic categories: high risk and non-high risk. The high risk group accounted for 63.75% of all closed claims and included claims related to chest pain, abdominal pain, wounds, fractures, pediatric fever/meningitis, central nervous system bleeding, abdominal aortic aneurysm, and epiglotitis. According to a study conducted in the Netherlands, 326 malpractice insurance claims were filed primarily for alleged errors in diagnosis and treatment [5]. 82% of claims involved minor surgical conditions, such as fracture, luxations, wounds and tendon injuries. Permanent disability was alleged in 22% of the claims and 4% involving death.

How risky is it for emergency physicians to care for emergency room (ER) patients in terms of the possibility of facing litigations in the courts? According to a U.S. government report, obstetrics/gynecology specialists were involved in the 12.4% of the claims closed in 1984 and ranked number one among all specialties; whereas emergency medicine specialty was only involved in 4.6% and ranked 8^th ^[6]. Childbirth negligence cases continue to be the area where physicians are losing the majority of cases that go to the jury in the U.S.; for instance, plaintiffs won 60% of these cases in 2002 [7].

However, since there are disproportionate numbers of obstetrics/gynecology patients and ER patients, the question then becomes how risky the ER patients are for emergency physicians in comparison with other patient populations. Most of the previous researches focus on physician level analyses. Our research aims at assessing the risks of encountering litigations in specified patient populations from an epidemiological perspective.

The purpose of this study is to assess the risk of being sued in the judicial system for caring ER patients from the perspective of epidemiology. Since birth in obstetrics has been considered the most risky practice in terms of professional liability, we compared with caring for giving birth to see relatively speaking how risky it is for emergency physicians to care for ER patients.

## Methods

This research was designed to be a retrospective cohort study based on secondary data analyses. We intended to find out the incidence of lawsuits arising from ER patients under emergency care and that of birth inpatients. The numbers of inpatients for each category of occurrences ended up in the district courts for each year were found out. The incidence rates were derived from dividing the numbers of specific lawsuits into the numbers of the predefined categories of patients for each year. Whether there is a difference among the mean incidences of the two groups were tested by Student's t test. The relative risks of developing litigation later on in each category of patients were computed by comparing the respective incidences.

In consideration of data availability, the study materials include the reimbursement claim dataset of the National Health Insurance (NHI) from 1998 to 2002, and the district court decision database of the Judicial Yuan from the August of 1999 to December of 2006. Taiwan has been able to accumulate a significant amount of computerized billing files since the initiation of the NHI in 1995. NHI started releasing their reimbursement database beginning from 1998. Since NHI is a mandatory health insurance, nearly 100% of all accidents and emergency, and births are treated in the NHI contracted health care providers. Therefore, we were able to ascertain the occurrences of all admissions of the desired parameters. All the cases that were reimbursed for emergency care were included in the ER patient population. All the cases that were reimbursed for birth were included in the birth inpatient population. The release of the NHI reimbursement claim dataset to be used in this study was approved by the National Health Research Institute, Taiwan.

Whether the case ends up in court was ascertained by looking into the district court decision database of the Judicial Yuan from the August of 1999 to December of 2006. The Judicial Yuan is Taiwan's judiciary and they started publishing court decisions since the August of 1999. We traced back to the year of the occurrence in the court's decision opinion. Only those fell into the 1998-2002 period were included in our analyses.

According to Florida's experiences on medical professional liability insurance claims, there is on average 1.2 years between occurrences and reporting [8]. It takes on average 75.16 days to close a criminal trial and 85.97 days to close a civil trial at the district court level in Taiwan in 2004 [9]. However, since the determination of most medical malpractice cases hinges on expert reports from the Department of Health (DOH), Taiwan, and the production of expert reports is time consuming, it usually takes more than one year to reach a judgment in either civil or criminal courts for medical malpractice lawsuits in Taiwan. Besides, there is no telling as to when the disgruntled patient or family will take the case to court so long as the statue of limitation has not run out. A two to three years' time lag between the occurrence of an unsatisfactory medical event and the first court judgment is a reasonable expectation according to the initial database exploration. Therefore, the available judicial database should be able to capture the occurrences that subsequently give rise to litigations to a satisfactory degree. If the same occurrence gave rise to more than one litigation, only one was counted.

## Results

In the years from 1998 to 2002, the annual inpatients of the whole nation average at two million and seven hundred thousands. Of the two million and seven hundred thousands, there are on average 251,894 births with an average cesarean section rate at 33.5%. On the other hand, hospital ERs see 6,065,620 patients annually. The descriptive statistics of the patient populations were summarized in Table 1.

**Table 1:** The descriptive statistics of target categories of patients by year

**Items**	**1998**	**1999**	**2000**	**2001**	**2002**	**Average**
Total ER patients	5,459,637	5,883,886	6,184,031	6,199,674	6,600,872	6,065,620
Total births	247,220	258,849	279,024	240,964	233,413	251,894
Natural birth	163,900	172,293	185,423	160,730	155,259	167,521
Cesarean section	83,320	86,556	93,601	80,234	78,154	84,373

After an extensive search of the Judicial Yuan database, there are 26 ER patients who later on sue their emergency physicians or their hospitals in the district courts in the entire study period (Table 2). Therefore, the average annual incidence rate of becoming a plaintiff among ER patients is 0.86 out of one million. Of the 26, 5 are not seriously injured, 2 seriously injured, and 19 dead. 7 are primarily treatment related and 19 diagnosis related. The principal diagnosis that accounted for the largest number of cases is traffic accident related injuries, followed by infection and acute myocardial infarction. 15 sued for civil damages and 11 sued for criminal convictions. Doctors lost in 8 cases (30.8%).

**Table 2:** The descriptive statistics of all lawsuits originated from ERs in 1998-2002^A^

**Items**	**N**	**%**
Total cases	26	100.0
Claim		
Diagnosis related	19	73.1
Treatment related	7	26.9
Final principal diagnosis		
Traffic Accident	10	38.5
*fracture*	*4*	*40.0*
*head injury*	*3*	*30.0*
*internal bleeding*	*2*	*20.0*
*intestinal perforation*	*1*	*10.0*
Infection and septic shock	4	15.4
Acute myocardial infarction	3	11.5
Iatrogenic	3	11.5
Intracranial hemorrhage	2	7.7
Rupture of abdominal aneurysm	1	3.8
Pulmonary edema	1	3.8
Adult respiratory distress syndrome	1	3.8
Congestive heart failure	1	3.8
Injury of the plaintiff		
Not seriously injured	5	19.2
Seriously injured	2	7.7
Dead	19	73.1
Defendant		
Emergency physicians^B^	23	88.5
Hospital	3	11.5
Judgment in the district court		
For plaintiff	8	30.8
Against plaintiff	18	69.2

^A^ER means emergency room.

^B^Emergency physician is defined as the physician that sees the plaintiff in the ER with unspecified specialty.

In contrast, there were total 42 birth related litigations from 1998 to 2002. 55% are criminal proceedings. The average annual incidence rate of becoming a plaintiff among birth patients is 33.5 out of one million. This incidence is significantly higher than that of ER patients (p < 0.01). For the detailed year by year breakdown please refer to Table 3.

**Table 3:** The descriptive statistics of litigations arising from target categories of patients by year

	**1998**	**1999**	**2000**	**2001**	**2002**	**Average**
Litigation related births	11	11	7	4	9	8.4
Criminal litigation related	7	7	4	2	3	4.6
Civil litigation related	4	4	3	2	6	3.8
Total births(× 10^5^)	2.47	2.59	2.79	2.41	2.33	2.52
Incidence of litigation related births(× 10^-5^)	4.45	4.25	2.51	1.66	3.86	3.35

Litigation related emergency patients	3	6	8	3	6	5.2
Criminal litigation related	3	6	2	0	0	2.2
Civil litigation related	0	0	6	3	6	3
Total ER patients(× 10^5^)	54.60	58.84	61.84	62.00	66.01	60.66
Incidence of litigation related ER patients(× 10^-5^)	0.06	0.10	0.13	0.05	0.09	0.09

Aside from respective incidences, the relative risk derived from comparing ER patients against births is 0.03, which means ER patients have 3% the risk of suing compared to birth inpatients, and this difference is statistically significant.

## Discussion

The problem of malpractice has threatened the development of health care around the world. For instance, in Japan, during the mid 1980s to the early 1990s, the total number of new medical malpractice lawsuits numbered in the three hundreds. However, in the late 1990s to the early 2000s, the number increased rapidly and reached the highest of 1,100 in 2004 alone [10]. On the other hand in the U.S., according to a health economics study, using a panel data set and a fixed-effects specification, the estimates indicate that malpractice litigation accounts for roughly 2-10% of medical expenditures, with the impact exceeding the dollar amount of settlements [11]. After the election of President Obama of the United States, the world has also been watching carefully about how the U.S. government goes about their health care reform. Experts predict that the Obama administration would have to make some compromises and bundle certain related initiatives together with the health care reform in order to make it happen, and medical liability reform is one of them [12]. Just like the trend in Japan and the U.S., there are more medical malpractice litigations over time and the awards are going up in Taiwan as well [7,13]. In comparing two mail surveys of physicians conducted in 1991 and 2005 respectively in Taiwan, malpractice claims in 2005 were more likely to be brought into courts than in 1991 (23.1% in 2005 vs 15.7% in 1991, odds ratio = 1.48) [14].

Similar to the Japanese [15-17] but not the American, physicians in Taiwan are likely to face both civil liabilities and criminal convictions in malpractice lawsuits. What is unique in Taiwan's situation is that most medical malpractice cases are tried in the criminal courts. Although the patients and their families have the choices between suing in the civil court directly or go to the prosecutors to seek an indictment, most plaintiffs opt for seeking an indictment for the sake of convenience and economy. The prosecutors have an obligation under Taiwan's Criminal Code to step in and investigate. If the physician is in the end convicted, he or she will face up to 5 years' imprisonment in the case of death claim and civil liabilities are also unavoidable [18]. Pursuant to Article 253-1 of Taiwan's Rules of Criminal Procedures [19], the prosecutor can initiate plea bargains with those who are charged of lesser crimes and likely to be indicted. Situations of physical injuries alleged in medical malpractice not resulting in death are considered to be lesser crimes. Therefore, the accused physicians can be offered the chances to plead guilty and face the kind of penalty they can accept, ranging from apologies to the patients, fines, social services, etc. Even if they are convicted, according to Article 74 of Taiwan's Criminal Code [18], defendants who receive the sentence of fewer than 2 years in prison can be put on probation for 2-5 years if they do not have previous criminal records. Most physicians will receive probation instead when they are found guilty of negligence in malpractice lawsuits. There are only very few instances that the accused physicians were thrown behind bars.

The other characteristic of our study is that we use the district level judgments to be the end point of our follow up through time. However, the litigation process is far from over. Most of the cases can still appeal to the appellate courts and the Supreme Court. Although most of the physicians in our study sample go free at the district level, the final verdicts are not in yet for a lot of them. However, for the purpose of this study, the beginning of their ordeals will give us an approximation of how risky their practices are.

Even if the absolute numbers of litigations did not give us the real picture of the numbers of malpractice disputes physicians are facing, the relative risk approach can serve as a good positioning beacon. Emergency physicians obviously face a lower risk patient population compared to obstetricians in terms of the propensity to sue in the judiciary; however, we have to bear in mind the fact that each emergency physician see a much larger volume of patients in comparison with obstetricians in delivery.

Unlike the study in the Netherlands where most of the claims involved minor surgical conditions and only 4% involved death, 73.1% of our lawsuits in the ER are death related [4]. This disparity further illustrated the difference between claims outside the legal system and those within. In the Netherlands' study, 79% of the study cases were closed within the medical liability insurer, which means only 21% of the cases remain unsettled and might end up in court. Combined our study results and theirs, we can draw the deduction that medical malpractice claims involving death are more difficult to settle out of court.

Although there is no definitive empirical evidence of defensive medicine [20], it has been widely recognized that physicians at high risk of litigation tend to practice defensive medicine. There are possibly two types of behaviors in defensive medicine: assurance and avoidance. Among the various possible assurance behaviors, physicians are most likely to order more CT, MRI or X ray; and in terms of avoidance behaviors, they are more likely to stop performing certain procedures than to avoid treating high-risk patients [1]. In comparison with other high risk specialties, emergency physicians are more likely to order diagnostic tests and less likely to stop performing specific procedures. From the information contained in Table 2, it appears that the most important allegation against emergency physicians in subsequent lawsuits is misdiagnosis. Therefore, it is quite possible that emergency physicians may order more laboratory tests, CTs, MRIs or X rays as assurance measures in facing ER patients.

In addition to the worry of practicing too much defensive medicine among specialists, we also wonder whether the increase of lawsuits will turn medical graduates away from choosing emergency medicine. According to a longitudinal study conducted in the U.S., medical malpractice concern did not affect the choice of emergency medicine residency among interns; however, this fear markedly decreases interns' enjoyment of medicine [21]. Whether this optimism holds true in other countries needs more observations in time.

Undoubtedly, a good portion of claims are either settled out of the judicial system or denied indictment. We will have to find the multiplier in order to approximate the real numbers of all malpractice disputes by inferring from the court decisions. If we can access the DOH's medical malpractice expert report files, we will have a better picture of how many disgruntled patients or families have sought the help of the prosecutors. Nonetheless, this approach still cannot help us find out how many claims have been settled without even going to the prosecutors. Commercial professional liability insurance has not been very popular in Taiwan so far. Most of the physicians simply joined the hospital's own self insurance program to protect themselves from huge malpractice payouts. The claim history of each hospital is one of their most guarded secrets. Therefore, there is no reliable claim history from the insurance industry, either. However, the same gap must have existed for the obstetricians.

Researchers of medical malpractice can approach this issue from two populations: physicians and patients. Most studies tend to center on physicians' behavior and characteristics. This research studied malpractice primarily based on the patient population. Similar studies although fewer are not uncommon. According to a hospital based case-control study conducted in Taiwan, the patients filing complaints with the hospital tend to come from the emergency room and surgical specialties, and live in the urban area; complaining patients who live in the urban area are more likely to file lawsuits eventually [22]. There are also studies trying to validate the connection between patient satisfaction surveys and malpractice suits. A study conducted in the U.S. indicates that, compared with physicians with the top satisfaction survey ratings, physicians in the middle tertile had malpractice lawsuit rates that were 26% higher, and physicians in the bottom tertile had malpractice lawsuit rates that were 110% higher [23]. Nonetheless, other experts have also cautioned that because lawsuits are infrequent events, calibrating these patient survey measures to malpractice lawsuit risks will require large physician samples from diverse practices [24]. By the same token, although this study covers a five year period, only 26 lawsuits are found and hence the generalizability of our findings is still limited. Besides, due to the limitation of data availability, this study only analyzed the NHI reimbursement claim data up to 2002 and the district court decision database up to 2006. Therefore, further studies based on longer and more updated observations are warranted to validate this study's findings.

## Conclusion

Emergency medicine, with its limited time for patient encounters, unpredictable flow, and lack of a continuing patient-physician relationship, is generally considered a high-risk specialty in terms of medical liability. Most malpractice related studies tend to center on physicians' behavior and characteristics. This research instead studied malpractice based on the patient population from an epidemiology's perspective. The study materials combined the reimbursement claim dataset of the NHI and the district court decision database. The average annual incidence rate of becoming a plaintiff for ER patients is 0.86 per million, and for birth patients is 33.5 per million. There is a statistically significant difference between birth patients and ER patients. The findings of our population based study indicate that the ER patient population emergency physicians are facing in Taiwan have relatively lower risks of developing litigation in comparison with the patients that come to give birth, 3% of birth patients' level of risk. That is to say, emergency doctors need to see almost 33 times more patients to reach the same level of risk facing obstetricians. Although the figure implies that emergency physicians are facing a patient population with lower risk in terms of filing medical malpractice lawsuits subsequently, nonetheless, we have to bear in mind that emergency physicians face a much larger volume of patients than obstetricians. Therefore, they can easily see 33 times more patients than their peers in obstetrics. Our findings also indicate misdiagnosis remains the major complaint of plaintiffs in subsequent litigations.

## Competing interests

The authors declare that they have no competing interests.

## Authors' contributions

CMY participated in study design, data collection and interpretation, performed the analyses and drafted the manuscript. SHT and WTC participated in the study design, interpretation and drafting of the manuscript. All authors read and approved the final manuscript.

## Pre-publication history

The pre-publication history for this paper can be accessed here:


